# The experience of healthcare workers to HIV pre-exposure prophylaxis (PrEP) implementation in low- and middle-income countries: a systematic review and qualitative meta-synthesis

**DOI:** 10.3389/fpubh.2023.1224461

**Published:** 2023-08-24

**Authors:** Liao Zhang, Yuqing Song, Xutong Zheng, Ying Liu, Hong Chen

**Affiliations:** ^1^West China Hospital, Sichuan University/West China School of Nursing, Sichuan University, Chengdu, China; ^2^School of Nursing, Chengdu University of Traditional Chinese Medicine, Chengdu, China; ^3^School of Nursing, Fujian University of Traditional Chinese Medicine, Fuzhou, China

**Keywords:** HIV, pre-exposure prophylaxis, healthcare workers, experience, low- and middle-income countries, meta-synthesis

## Abstract

**Background:**

The effectiveness of pre-exposure prophylaxis has been extensively documented. However, there are substantial gaps between the actual implementation of pre-exposure prophylaxis and the ideal goal, especially in low-and middle-income countries. Healthcare workers play critical roles in the pre-exposure prophylaxis implementation, and they have more multi-level experiences about the barriers of pre-exposure prophylaxis implementation and how to facilitate it. However, the evidence aiming to synthesize their experiences is limited.

**Objective:**

This study aims to aggregate the healthcare workers’ experiences of providing pre-exposure prophylaxis in low-and middle-income countries, and find the barriers, facilitators, and recommendations of pre-exposure prophylaxis implementation.

**Methods:**

The ENTREQ (Enhancing transparency in reporting the synthesis of qualitative research) statement was used to guide the design and reporting of this qualitative meta-synthesis. A comprehensive search was conducted from inception of databases to 16^th^ March 2023 in four databases: PubMed, CINAHL Plus with Full Text, Embase, Web of Science. The quality appraisal was conducted using the Joanna Briggs Institute Critical Appraisal Checklist. JBI’s meta-aggregation approach was used to guide the data extraction and synthesis, and the JBI ConQual approach was used to evaluate the evidence level of the synthesized findings.

**Results:**

Fourteen articles with good methodological quality were included in this review. A total of 122 findings were extracted and 117 findings with credibility ratings of “unequivocal” or “equivocal” were included in this meta-synthesis. The eligible findings were aggregated into 13 new categories and subsequently developed into 3 synthesized findings: the barriers, facilitators, and recommendations of pre-exposure prophylaxis implementation in low-and middle-income countries. The overall ConQual score of all three synthesized findings was rated as “low.”

**Conclusion:**

This review aggregated the experience of health care workers implementing pre-exposure prophylaxis in low-and middle-income countries and we could focus on the following key points to promote the uptake of pre-exposure prophylaxis: improve knowledge about pre-exposure prophylaxis, create a supportive environment, address medication-related barriers, increase the human resources and financial investments, and diversify the providing models.

**Systematic review registration:**

https://www.crd.york.ac.uk/PROSPERO/. The protocol of this review has been registered in the International Prospective Register of Systematic Reviews (PROSPERO, CRD42023411604).

## 1. Introduction

Despite substantial progress made in the control of the human immunodeficiency virus (HIV) epidemic worldwide, there were still an estimated 1.5 million new infections in 2021. HIV remains a major global health issue (1). Especially in low-and middle-income countries (LMICs), because of HIV-related stigma, exposure to stressful life events, poverty, and limited access to health care services, the HIV epidemic is particularly severe (2). For instance, the LMICs in Sub-Saharan Africa which accounted for over two-thirds of the global HIV burden (25.6 million) (3). The regions with high prevalence of HIV particularly in LMICs constitute a challenge to ending the HIV pandemic worldwide.

To prevent the transmission of HIV, a series of biomedical HIV prevention technologies have been developed and rapidly scaled up, such as voluntary male medical circumcision, treatment as prevention, oral pre-exposure prophylaxis (PrEP), post-exposure prophylaxis (PEP), prevention of mother-to-child transmission, and HIV testing and counseling (4). PEP is the administration of antiretroviral therapy to an HIV-negative person who may expose to HIV, while PrEP is the use of antiretroviral drugs in a high-risk, HIV-negative patient, to prevent future HIV infections, and PrEP was focused in this review. The first PrEP approved by the US Food and Drug Administration (FDA), Truvada, is a combination of two antiretroviral drugs, oral tenofovir disproxil fumarate and emtricitabine (TDF/FTC), whose benefits for HIV prevention have been well proven (5, 6). Clinical trials have demonstrated that consistent use of PrEP can reduce the risk of HIV acquisition by over 90%, making it one of the most important biomedical strategies in HIV prevention (7, 8). The World Health Organization (WHO) has expanded its recommendations on who is eligible for PrEP from key populations to all people at significant risk of HIV infection (9). Consequently, PrEP now forms an important part of the HIV prevention approach available to populations in settings with high HIV prevalence.

Considering the broad evidence of the effectiveness, acceptability, and feasibility of PrEP, the number of countries implementing PrEP has been increasing over the last few years, and over 90 countries have approved the use of PrEP for HIV prevention as of December 2022 (10). However, there are substantial gaps in the availability of PrEP across different counties, and much of the expansion of PrEP remains highly concentrated in a rather small number of countries, notably the United States of America. In 2020, the total number of people in LMICs using this prevention option was only 28% of the target of 3 million and only 8% of the new global 2025 target (11).

Previous reviews (12–14) have explored the gap between the actual implementation of PrEP and the ideal goal, and the related barriers and facilitators were excavated from different perspectives, the most reported factors were lack of PrEP awareness, knowledge, and willingness. However, the current research and reviews have mostly concentrated on high-income countries and the perspectives of the target populations, insufficient attention has been given to LMICs and the perspectives of healthcare workers (HCWs). HCWs play critical roles in the successful implementation of PrEP, and possess valuable multilevel experiences regarding barriers and facilitator (15). On the other hand, as ‘gatekeepers’, HCWs are need to be informed about PrEP management, be willing to prescribe it, and need to be supported with resources to foster this novel intervention into practice. However, the progress on HCWs-initiated PrEP has been less than ideal, suggesting that it is imperative to understand their attitudes, perceptions, and perceived barriers to PrEP implementation, for motivating them to prescribe PrEP and providing them with the necessary support (16). Interviews with HCWs based on qualitative research methods can provide richness of evidence to address clinical practice or policy-related barriers about PrEP implementation. Some qualitative studies have identified the barriers, facilitators, and recommendations for PrEP implementation in LMICs from the perspective of HCWs. However, several qualitative studies only assessed the experience in selected key populations, such as adolescent girls and young women (AGYW) (4, 17, 18), pregnant and postpartum women (15), or fisherfolk (19), making it challenging to form a reference to guide the implementation of PrEP for all kinds of populations in LMICs.

Meta-aggregation approach was used in this review to synthesize the results of existing qualitative studies on the barriers, facilitators, and recommendations of PrEP implementation from the perspectives of HCWs in LMICs. Mata-aggregation is grounded in the philosophic traditions of pragmatism and Husserlian transcendental phenomenology, and it is usually used to produce recommendations to guide practitioners and policymakers (20). And the objective of this review was to aggregate the HCWs’ experiences of providing PrEP to different target groups and in different regions in LMICs and provide practical recommendations for future practice and research.

## 2. Methods

### 2.1. Design

This meta-synthesis of qualitative studies was conducted to explore the experience of PrEP implementation in low-and middle-income countries from the perspective of HCWs and to answer the following three questions: what are the barriers to PrEP implementation? What factors can facilitate PrEP scale-up? Based on the current situation and experience, what recommendations could improve the PrEP implementation? The ENTREQ (Enhancing transparency in reporting the synthesis of qualitative research) statement was used to guide the design and reporting of this qualitative meta-synthesis (21). This review was registered with International Prospective Register of Systematic Reviews (PROSPERO, CRD42023411604).

### 2.2. Search strategy

A comprehensive search was conducted from the databases inception to 16^th^ March 2023 in four databases, PubMed, CINAHL Plus with Full Text, Embase, and Web of Science. The search strategy used a combination of medical subject headings (MeSH), title, abstract, keywords and Boolean calculation for capturing the three key concepts: (1) HIV, (2) PrEP, and (3) qualitative study. Additionally, we manually searched through the reference list of the included articles and relevant reviews published previously. To avoid omitting potentially relevant studies, we did not limit the participant and country of the articles during the process of searching. The details of all search strategies we used are available in Supplementary Material 1.

### 2.3. Eligibility criteria

The primary studies were selected following the PICoS format (participants, phenomenon of interest, context, and study design). Studies were included if they met all the following criteria: (1) Participants: For the purpose of this review, HCWs were broadly defined to doctors, nurses, pharmacists, clinical coordinators, healthcare providers, social workers, counselors, community staff, PrEP program managers, and other providers who were directly or potentially involved in PrEP implementation. (2) Phenomenon of interest: the (potential) barriers and facilitators during PrEP implementation, and recommendations improving PrEP implementation based on their experience and cognition. (3) Context: We limited the study settings to low-and middle-income countries following World Bank Country and Lending Groups (22). (4) Study design: qualitative research with no limitation of the methodology (e.g., phenomenology, ethnography, or grounded theory method) and the mixed method studies were included if they offered clear qualitative analysis and the primary data could be extracted. Exclusion criteria included: (1) Review articles, conference abstracts, posters, books, and dissertations, (2) The participants were HCWs plus other populations, which made it difficult to separately extract the qualitative data of HCWs separately, (3) Repeated publications, (4) Unavailable full texts, and (5) Non-English articles.

### 2.4. Study selection

**Figure 1:**
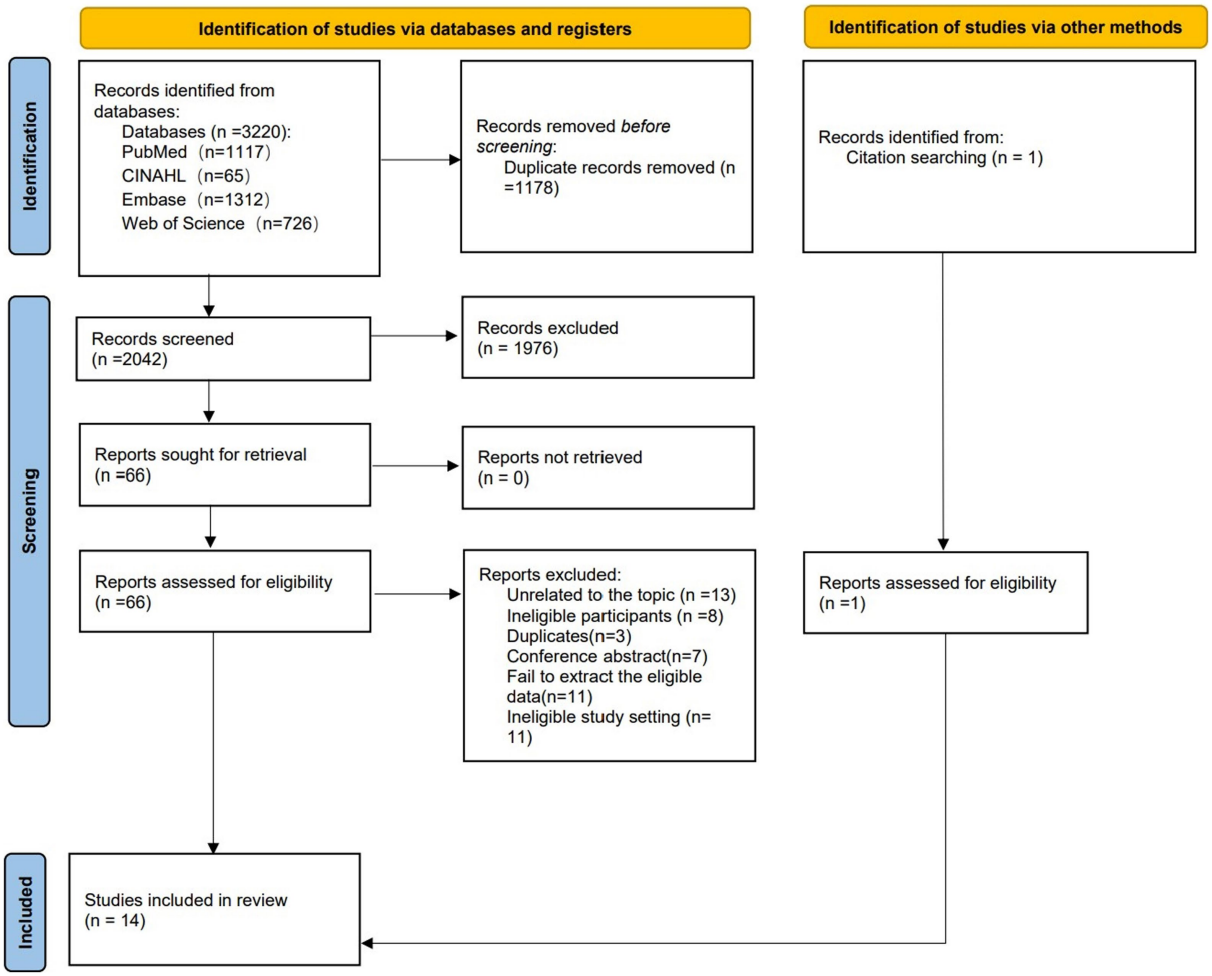
PRISMA flowchart.

The process of selection was performed by two authors independently (Zhang and Liu) independently, following the PRISMA guidelines (see Figure.1). All search results were imported into the reference management program Endnote X9. After removing duplicates from the primary 3,220 studies, the two researchers independently screened the titles and abstracts of the studies following our inclusion criteria. We read the full text of potentially relevant studies to select the eligible articles to be included in this review and meta-synthesis, and we detailed and categorized the reasons for the exclusion of the excluded studies. Any disagreement in the selection process was resolved through a discussion between two researchers or consultation with a third researcher (Chen).

### 2.5. Quality appraisal

The quality appraisal was independently conducted by the same two researchers (Zhang and Liu) following the Joanna Briggs Institute Critical Appraisal Checklist for critical and interpretive research (23). This checklist consisted of 10 items (e.g., is there congruity between the stated philosophical perspective and the research methodology?), and each item was scored as “yes,” “no” or “not applicable,” we need to make an overall appraisal with “include,” “exclude” or “seek further info” after evaluating all items. If 60% of the items answered “yes,” the quality of the study was considered acceptable, 70–90% answered “yes” to indicate good quality, and if 100% of the items answered “yes,” the quality of the study was considered high. A study was included if the items achieved a minimum of 60% “yes.”

### 2.6. Date extraction and synthesis

JBI’s meta-aggregation approach was used to guide the data extraction and synthesis. This approach is grounded in the philosophic traditions of pragmatism and Husserlian transcendental phenomenology. It was a widely used method with the pragmatic aim of systematically reviewing qualitative research to generate synthesized findings that can be used to inform healthcare practice or policy, which was perfectly aligned with the purpose of this review (23).

The data extraction involved two phases: (1) extracting the general details of the study, such as citation details, and (2) extracting findings, which were defined as verbatim extracts of the author’s analytic interpretation of the results or data. During the extraction of findings, the level of “credibility” should be allocated based on the reviewers’ assessment of the degree of fit, or congruency between the data and the accompanying exemplar quote. Three levels of credibility were used, “unequivocal” if the congruence was beyond a reasonable doubt, “equivocal” if a clear association was lacking, and “unsupported” if the data did not support the findings. Only unequivocal and equivocal findings were included, and unsupported findings were not presented in the synthesis result (23). The specific process of data extraction was conducted by the same two researchers using a predesigned Excel spreadsheet, including the first author, publication year, country, type of participants, study setting, study aim, sample size, methodology, sampling approach, method of data collection and analysis, major themes, subthemes, and primary quotes. The extracted information was verified by a third researcher (Chen), and any disagreement was resolved through discussion among three authors (Zhang, Liu, and Chen).

The data synthesis followed a three-step approach. Step 1, extracting of findings from included studies (this is the second phase of the data extraction as well). Step 2, pooling the findings into new categories based on the similarity in meaning (each category consisted of at least two findings). Step 3, developing one or more synthesized statements of at least two categories.

### 2.7. Quality appraisal of each synthesized finding

The JBI ConQual approach was used to evaluate the quality of synthesized findings, which were ranked via a downgraded system based on the dependability of the included studies and the credibility of the findings (24). Five items from the JBI critical appraisal checklist (item No 2, 3, 4, 6, and 7) were used to assess dependability. Dependability was rated high if 4–5 items were appraised with “yes,” moderate if 2–3 items were appraised with “yes,” and low if 0–1 items were appraised with “yes.” The credibility was evaluated as unequivocal, equivocal, and unsupported. If most included studies in a synthesized finding had a dependability rating of high/moderate/low, the dependability of the synthesized finding remained “high”/degraded 1 level/degraded 2 levels. The overall credibility of a synthesized finding remained “high” if it was consisted of unequivocal findings, degraded 1 level if it consisted of a mixture of unequivocal and equivocal findings, degraded 2 levels if it consisted of equivocal findings, degraded 3 levels if it consisted of a mixture of equivocal and unsupported findings, and degraded 4 levels if it consisted of unsupported findings. The overall ConQual score was rated with “high,” “moderate,” “low,” and “very low,” and started with “high” and was downgraded one level for every downgrade in the dependability and credibility scores.

## 3. Result

### 3.1. Searching results

A total of 3,220 records were identified from 4 databases and 1 from other sources. After removing duplicates, 2042 records remained. These remaining records were screened by titles and abstracts, resulting in the exclusion of 1976 articles. Subsequently, the full texts of 66 articles were retrieved to assess eligibility. Finally, 14 articles were included in this review. The PRISMA flowchart in Figure 1 presents the selection pathway for the final inclusion of the studies.

### 3.2. Study characteristics

All 14 included studies (4, 15, 17–19, 25–33) were published between 2014 and 2023 in seven low-and middle-income countries, including Kenya (*n* = 6), Tanzania (*n* = 1), Eswatini (*n* = 1), Philippines (*n* = 1), Ugandan (*n* = 1), South Africa (*n* = 1), Zimbabwe (*n* = 1), and multi-center studies (*n* = 2, one study was conducted in Kenya, South Africa, and Zimbabwe; the other one was conducted in Kenya and Uganda). Qualitative study design without a specific method was used in most of the included studies (*n* = 12, 85.72%). The specific type of HCWs varied greatly across the studies, and 49 primary themes relating to our purpose were reported in the 14 included studies. The details of the included studies are shown in Table 1.

**Table 1:** Study characteristics.

Study	Country	Participant	Setting	Aim	Sample size	Methodologic and sampling approach	Method of data collection and analysis	Major theme
Duby (2023)	South Africa	Program managers, project coordinators, HCPs/ nurses, social workers, counsellors, peer group trainers and outreach workers	Telephone	To explore PrEP implementation experiences of AGYW	38	Qualitative design Purposive sampling	Semi-structured interview Immersive iterative thematic analysis	PrEP demand creation and acceptability of PrEP amongst AGYW Community acceptability of PrEP Parental acceptability of PrEP The need to address barriers towards PrEP acceptability Successful strategies for increasing PrEP acceptability
Skovdal (2022)	Zimbabwe	Nurses	A private room at health facilities	To explores the views and recommendations on how to increase uptake of PrEP.	12	Qualitative design Purposive sampling	Semi-structured topic interview Thematic analysis	Strengthen the human resource capacity Improvements in PrEP treatment and delivery Make available youth-friendly PrEP services Need for PrEP awareness campaigns Engage AGYW Eliminate costs associated with PrEP uptake
Omollo (2022)	Kenya	HCPs	Face-to-face in a private room / phone call	To understand barriers, facilitators and strategies to PrEP implementation	15	Qualitative design Purposive sampling	Semi-structured IDI Thematic content analysis.	Friendly and non-judgmental tones build AGYW trust to discuss PrEP Confidentiality is key Active listening and tailored counselling Supporting client agency promotes AGYW empowerment and effective PrEP use
Mwongeli (2020)	Kenya	Clinical officers, nurses, nurse counselors, mentor mothers, pharmacists, community health workers and psychologists	A private room at the clinic	To identify the acceptability and feasibility of PrEP implementation among Kenyan HCWs	45	Qualitative design Purposive sampling	Semi-structured interviews Conventional content analysis	Knowledge gaps regarding eligibility and risk assessment and HCW attitudes might limit PrEP implementation Multiple facility and interpersonal level barriers of PrEP implementation
Kimani (2022)	Kenya	Clinicians, nurses, administrators, and records officers	A private space in the hospital	To explore the opinions of HCP on perceived and actual barriers to PrEP programming.	11	Qualitative design Maximum variation purposive sampling	Focus group discussions Thematic approach	PrEP provision preparedness Recommendations to improve PrEP programming and reach
Camlin (2022)	Kenya and Uganda	Clinical officers, nurses, community-based nurse trackers, PrEP ambassadors	NR	To explore providers’ attitudes and experiences with PrEP delivery To understand the barriers and facilitators encountered by providers	19	Qualitative study Purposive sample	Semi-structured IDIs Interpretivist approaches	Heterogenous attitudes toward PrEP Concerns about patient behavior and provider responsibility Communication challenges Concerns regarding serving specific client populations Support for sero different couples Continuation facilitators
Bogart (2022)	Ugandan	HCPs, policymakers, community leaders	A private space	To identify feasible strategies to improve PrEP implementation and social marketing messages to encourage PrEP use	10	Qualitative design Purposive sampling	In-person semi-structured interviews A directed content analysis approach	Barriers to PrEP Implementation Recommendations for PrEP Implementation
Roche (2021)	Kenya	PrEP providers	A private room at the pharmacy	To understand factors that may influence the ability or willingness to uptake or deliver PrEP at retail pharmacies	10	Qualitative design Purposive sampling	Semi-structured interview Conventional content analysis	Anticipated relative advantages of pharmacy-based versus clinic-based PrEP delivery Determinants of acceptability of pharmacy-based PrEP delivery Determinants of feasibility of pharmacy-based PrEP delivery
Restar (2021)	Philippines	Medical doctors, nurses, clinic coordinators, counselors, and staff volunteers	In the key informants’ local offices	To examine and explore awareness and perceptions of HCWs on implementing PrEP and PEP programs	15	Phenomenological approach Snowball sampling	Semi-structured interview Inductive thematic analysis	Lack of knowledge of PrEP Logistical issues surrounding PrEP guidelines Equity in funding and preferences for HIV treatment vs. prevention Risk compensation
Lanham (2021)	Kenya, South Africa, and Zimbabwe	Clinician Nurse, counselor, and community-based worker	Private locations convenient to participants	To summarize the providers’ attitudes and experiences of delivering PrEP to AGYM	113	Qualitative study Extreme case sampling	IDI Thematic analysis	Uptake, adherence, and continuation challenges Service delivery strategies
Jackson-Gibson (2021)	Kenya	Organization staff, health care providers and community leaders	NR	To identify facilitators and barriers influencing implementation outcomes	15	Qualitative design Purposeful sampling	KIIs Thematic content analysis	Facilitators to PrEP implementation, uptake and persistence Barriers to PrEP uptake and persistence
Bärnighausen (2020)	Eswatini	Nurse, expert client, counsellor nursing assistant, mentor mother	NR	To examines barriers and facilitators to PrEP uptake and adherence among the general population	26	Grounded Theory Purposive sample	Semi-structured IDIs Three phases of coding	Barriers of PrEP implementation Facilitators of PrEP implementation Recommendations of PrEP implementation
Pilgrim (2018)	Tanzania	Nurse, doctor, clinical, officer, counselor	NR	To examine HCPs’ knowledge, attitude, and skills, as well as their perceptions of facility readiness to provide PrEP	24	Qualitative study Purposive sample	Semi-structured IDI Thematic content analysis	Provider-level perspectives on PrEP Facility-level perspectives on PrEP
Mack (2014)	Kenya	Community stakeholders	A hotel in Bondo	To collect experience from community stakeholder to provide reference for future PrEP implementation	20	Qualitative design NR	Focus consultation Iterative analysis	Service delivery-level experience Community-level experience Target population-level experience Lifestyle and circumstances of target populations Fears, lack of awareness, and misinformation among target populations Issues for uptake of PrEP among target populations

HCP = Health Care Provider PrEP = Pre-Exposure Prophylaxis AGYW = Adolescent Girls and Young Women IDI = in-depth interview HCW=Health Care Worker NR = Not Report KII = key informant interview.

### 3.3. Methodological quality

All 14 included studies had a good level of methodological quality, as shown in Table 2. All studies did not state their philosophical perspective, leading to an unclear congruity between it and the research methodology. Only one study (33) located the researcher culturally, and four studies (18, 29, 30, 33) reported how to address the influence of the researcher on research and *vice-versa*. For the remaining seven items, all studies met the “yes” criteria.

**Table 2:** Methodological quality.

Study	1	2	3	4	5	6	7	8	9	10	Total percent of “yes”	Overall quality
Duby (2023)	U	Y	Y	Y	Y	N	U	Y	Y	Y	70%	Good
Skovdal (2022)	U	Y	Y	Y	Y	N	U	Y	Y	Y	70%	Good
Omollo (2022)	U	Y	Y	Y	Y	N	Y	Y	Y	Y	80%	Good
Mwongeli (2020)	U	Y	Y	Y	Y	N	U	Y	Y	Y	70%	Good
Kimani (2022)	U	Y	Y	Y	Y	Y	Y	Y	Y	Y	90%	Good
Camlin (2022)	U	Y	Y	Y	Y	N	N	Y	Y	Y	70%	Good
Bogart (2022)	U	Y	Y	Y	Y	N	N	Y	Y	Y	70%	Good
Roche (2021)	U	Y	Y	Y	Y	N	N	Y	Y	Y	70%	Good
Restar (2021)	U	Y	Y	Y	Y	N	Y	Y	Y	Y	80%	Good
Lanham (2021)	U	Y	Y	Y	Y	N	Y	Y	Y	Y	80%	Good
Jackson-Gibson (2021)	U	Y	Y	Y	Y	N	N	Y	Y	Y	70%	Good
Bärnighausen (2020)	U	Y	Y	Y	Y	N	N	Y	Y	Y	70%	Good
Pilgrim (2018)	U	Y	Y	Y	Y	N	N	Y	Y	Y	70%	Good
Mack (2014)	U	Y	Y	Y	Y	N	N	Y	Y	Y	70%	Good

1. Is there congruity between the stated philosophical perspective and the research method? 2. Is there congruity between the research methodology and the research question or objectives? 3. Is there congruity between the research methodology and the methods used to collect data? 4. Is there congruity between the research methodology and the representation and analysis of data? 5. Is there congruity between the research methodology and the interpretation of results? 6. Is there a statement locating the researcher culturally or theoretically? 7. Is the influence of the researcher on the research, and vice-versa, addressed? 8. Are participants, and their voices, adequately represented? 9. Is the research ethical according to current criteria or, for recent studies, and is there evidence of ethical approval by an appropriate body? 10. Do the conclusions drawn in the research report flow from the analysis, or interpretation, of the data?

Y = Yes, N = No, U=Unclear.

### 3.4. Meta-aggregation

A total of 122 findings were extracted from the 14 included studies. We included a total of 117 finding with credibility rated unequivocal or equivocal in this meta-synthesis, and 5 findings with “unsupported” plausibility were excluded. The 117 eligible findings were aggregated into 13 new categories and subsequently developed into 3 synthesized findings: the barriers, facilitators, and recommendations. Full details of the credibility evaluation process and findings synthesis could be found in Supplementary Material 2 and 3.

#### 3.4.1. Synthesized finding 1: the barriers to PrEP implementation

From the statements of HCWs, we emerged five categories of barriers to PrEP implementation, medication-related barriers, stigma towards PrEP, barriers at the level of providers and facility, misinformation about PrEP, and the cost of service obtaining.

##### 3.4.1.1. Medication-related barriers

Many target populations may hesitate to take PrEP due to the fear of the negative side-effects, such as getting a rash or insomnia. The adherence to daily prevention pills was also a big challenge of the PrEP implementation. They may also refuse to start and continue PrEP because of related testing, for example, being pricked to take a blood test.

Some would say that they get a rash, … cannot sleep… they will go around saying “these pills!”… So, most of them (AGYW) will say, “no, I do not want it, ….” [from a service provider, (17), p. 137].

They (AGYW) do not want PrEP, … because they feel like they are eating treatment (ARVs) every day and yet they are not HIV positive, … they wish it was an injection as opposed to taking pills every day. [from a social worker, (17), p. 137].

##### 3.4.1.2. Stigma towards PrEP

There were prevalent misconceptions about PrEP in the community, they held that the PrEP was an antiretroviral and used for HIV treatment, causing the target populations may refuse to take PrEP for fear of being perceived as an HIV-positive person. In addition, they may link the PrEP to risky sexual behaviors, such as condomless sex, earlier sexual initiation, and having more sexual partners. Consequently, the social stigma towards PrEP have become significant barriers.

“oh, this is ARV”…“Why are you taking HIV medications?”… they end up getting stigmatized and being accused of having HIV… so that makes them (AGYW) very scared. “my parents will think I’ve got HIV.” [from a health care provider, (17), p137].

##### 3.4.1.3. Barriers at the level of providers and facilities

The lack of human and financial resources was an important challenge for the healthcare institutions. Starting a PrEP service required additional staff and testing equipment, thereby adding strain to current healthcare system. As for healthcare providers, some of them expressed the uncertain effectiveness of PrEP to prevent HIV, and they did not know how and when to provide PrEP because of the lack of current information on PrEP studies as well as prescribing recommendations and guidelines, which was a potential barrier to implementation of PrEP.

“Staffing is an issue, cause at times … you are alone here [at clinic]. So, you are left with your hands tied… So maybe I can say staffing issue is a challenge.” [from a healthcare provider, (28), p. 9].

There’s no… large-scale studies …that would recommend [PrEP] yet… If it does prevent HIV, I hope that … there would be clear recommendation and guidelines regarding when to use it. [from a medical doctor, (30), p. 676].

##### 3.4.1.4. Misinformation about PrEP

There were three main types of misinformation about PrEP. First, they did not know the role of PrEP and failed to differentiate between PrEP for the prevention of HIV infection and antiretrovirals for the treatment of HIV, which may generate misunderstanding and even family conflicts and eventually lead to PrEP discontinuation. Second, there were misconceptions about the side effects of PrEP, with some believing that it can cause infertility, leading to the termination of pregnancy or even an increased risk of HIV infection. The third misinformation was that they established an inevitable and unreasonable connection between PrEP uptake and sexual promiscuity, suggesting that people who take PrEP increase risk behaviors, engage in unprotected sex, and lead to unwanted pregnancies and sexually transmitted diseases.

AGYW would say they were told that PrEP is like ARVs, which is due to lack of information about PrEP.[from a clinic-based implementer, (17), p136].

Some of them complain of side effects even when they have not even started taking the pills! [from a nurse tracker, (32), p399].

People may become careless and get involved in sexual activities knowing that there is a preventive measure for HIV. …I think there would be an increase of other sexual transmitted infections as most people do not fear syphilis or gonorrhea. [from a clinical officer, (26), p10].

##### 3.4.1.5. The cost of service obtaining

The major cost came from the following three sections, the transport fee to the dispensing facilities, the cost of pills, and the monitoring expenses. Before and during PrEP implementation, a lot of laboratory tests were needed to confirm their acquisition qualification, effectiveness and side effects of the PrEP. Many target populations may fail to afford the cost.

Yes, they would have a lot of follow ups…monitoring if they have [PrEP]…It takes a lot of laboratory monitoring,… Some of our client cannot afford that. [from a counselor in clinic, (30), p. 677].

#### 3.4.2. Synthesized finding 2: the facilitators of PrEP implementation

Three categories emerged about the facilitators of PrEP implementation, creating a supportive environment, the positive experience of service acquisition, and perceived benefit of PrEP.

##### 3.4.2.1. Creating a supportive environment

The initiation and adherence of PrEP uptake could be improved by encouraging parents and sexual partner(s) to positively engage in PrEP promotion and education. Their perceptions were expected to change by obtaining more information about PrEP, and a conducive and supportive environment was created, which was an essential step in the implementation strategy.

But through more engagement, parents are more understanding and some parents are even calling us to initiate PrEP for their kids. [from a health care provider, (17), p141].

…If the wife say ‘I’m now using PrEP’ it because the husband already has the information. [from a community staff, (28), p7].

##### 3.4.2.2. The positive experience of service acquisition

A positive experience of service acquisition was a key factor in determining clients’ initiation of and adherence to PrEP. Many target populations reported strong previsit anxiety and fear of being labeled as sexually active. To establish a successful client-provider relationship, a non-judgmental tone to clients’ sexual activity and demands was needed in the context of delivering PrEP. Meanwhile, keeping the information confidential and providing a safe space were essential in building trust with clients.

“We always ensure that we tell these clients that there is paramount privacy and confidentiality. …a special space where they can be talked to, where they feel their issues are private and confidential. So, it is a safe place for these young women.” [from a healthcare provider, (18), pp7-8].

##### 3.4.2.3. Perceived benefit of PrEP

The clients’ and providers’ enthusiasm for PrEP is a key facilitator of PrEP implementation. Particularly, among sero different couples, daily PrEP-taking could facilitate relationship harmony and protection from HIV. After the encounter, they did not have to keep wondering if they were infected, and they did not have to be stressed over that.

…it helps to protect the negative partner from seroconverting. …One participant came the other day and told me, ‘My husband now is very happy because I am also taking PrEP. He is now motivated.’ [from a nurse tracker, (32), p. 400].

Our main program entails offering PrEP to HIV negative girls… there was excitement for the fact that now these girls have a chance of being the future generation of HIV negative. [from a health care provider， (17), p. 142].

#### 3.4.3. Synthesized finding 3: recommendations to facilitate PrEP implementation

Five categories emerged about the facilitators of PrEP implementation: finding support, changing medication, improving the perceptions about PrEP, increasing human and financial investment, and optimizing the current PrEP providing service model.

##### 3.4.3.1. Finding support

One recommendation to improve PrEP uptake was to build a supportive relationship between clients and their parents or partners. With support from their close contacts, the target population would be more willing to initiate PrEP. Their supporters could keep reminding them to take pills to improve their adherence to PrEP. Some active peers who have initiated PrEP could also be recruited as “ambassadors” to encourage and support more potential target peers to join the PrEP program.

They need to get people who can at least remind them, or whom we can call their treatment supporters. [from a healthcare provider, (19), p6].

so what we have decided to do is to use their own peers that are on PrEP already, to say “you are PrEP ambassadors, go and recruit your own peers to be initiated on PrEP.” [from a program manager, (17), p. 138].

##### 3.4.3.2. Changing medication

HCWs held that the clients’ anticipation of side effects may constitute a significant worry and deterrent to engaging with PrEP. HCWS should dispel rumors about side effects and inform clients about how to reduce and address them. Additionally, minimizing the size of pills or changing the oral pills to a long-acting injectable PrEP to address the poor adherence to daily medication. Meanwhile, dedicated packaging and clear labels could be used to indicate that medication is for HIV prevention instead of treatment, thus, PrEP related stigma may be reduced.

PrEP should be dispensed in another container. They do not want it to be dispensed in its original containers which are easily identified and associated with HIV treatment. [from a healthcare provider, (4), p5].

they wish it was an injection as opposed to taking pills every day. [from a social worker, (17), p137].

Even when you hold it in your hands. It’s too big. [from a healthcare provider, (4), p4].

##### 3.4.3.3. Improving the perceptions about PrEP

Three key groups should be focused on for the perception improvement of PrEP, healthcare workers, target populations and their close contacts. For HCWs, regular training is needed to ensure that they master the information about PrEP and can provide optimal HIV prevention and treatment services. To improve the perceptions about PrEP of target populations and their close contacts, some PrEP awareness campaigns could be carried out in the community and in schools or via other preferred media. Another key point was that we should conduct comprehensive education patiently before and after the target population initiates the PrEP.

Campaigns about PrEP should be done in the community and in schools. …this will help us in HIV prevention. [form a healthcare provider, (4), p5].

Yes, we will need training …the staff need to be updated frequently. Although they are all trained personnel, some may neglect a few things and they need to get a refresher course training in certain areas to be aware of new things. [from a clinical officer, (26), p. 12].

##### 3.4.3.4. Increasing human and financial investment

There was a general perception among healthcare providers that their human resource capacity was inadequate. More staff should be recruited and trained with professional courses to make available the time required to counsel and support clients seeking PrEP instead of having part-time HCWs squeeze in the time to provide these services. On the other hand, there are a considerable number of potential clients from poor areas or students with no income, and they are unable to afford the PrEP or even the transport fares. More financial resources should be invested to address the cost-related barriers to PrEP uptake. Furthermore, appropriate monetary incentives or material goods could be used to promote the PrEP implementation.

…There is need to have someone dedicated to PrEP and nothing else. For example, someone like me may be busy helping someone deliver their baby, delaying someone seeking PrEP. When someone gets up and decides to look for PrEP, it would be helpful for them to get the services right away. [from a healthcare worker, (4), p. 3].

##### 3.4.3.5. Optimizing the current PrEP providing service model

The current PrEP providing service model could be optimized in two ways. First, we could change the location of service delivery and integrate PrEP counseling and delivery services into the current health system, such as obtaining drugs in general clinics rather than sexual health clinics, which may reduce the potential stigma. We can also deliver drugs through public pharmacies or communities. Second, by changing the traditional offline service model, we could use online media to conduct promoting campaigns, provide consulting services for clients and remind them to promote their adherence.

I think PrEP should have taken a different route like may be dispensed at the outpatient or integrated into public health but not here. [from a healthcare provider, (33), p. 292].

…I can give out to communities, they could give us a number of different types of drugs that treat all diseases. So, in the process of giving out these drugs to people we could give out PrEP refills as well. …. [from a community health worker volunteer, (19), p. 7].

I make a follow up using WhatsApp, and if I cannot get hold of her, I ask the peer counselors to make a follow up. [from a nurse providing PrEP, (29), p. 7].

### 3.5. Quality appraisal of synthesized findings

The quality appraisal of the synthesized findings is presented in Table 3. The overall ConQual score of all three synthesized findings was rated as “low.”

**Table 3:** Quality appraisal of synthesized findings.

Synthesized finding	Dependability	Credibility	ConQual score
The barriers of PrEP implementation	Downgrade1 level	Downgrade1 level	Low
The facilitators of PrEP implementation	Downgrade1 level	Downgrade1 level	Low
Recommendations to facilitate PrEP implementation	Downgrade1 level	Downgrade1 level	Low

## 4. Discussion

To the best of our knowledge, this article was the first systematic review and meta-synthesis of qualitative studies on the experience of HCWs in providing PrEP in different regions of LMICs following the meta-aggregation approach. It demonstrated the barriers faced in PrEP initiation and persistence, the successful promotion strategies in the implementation of PrEP, and valuable recommendations to facilitate PrEP scale-up in LMICs. This review found the gap between the actual situation and the target of PrEP implementation in LMICs, and highlighted the importance of the experience of HCWs in PrEP delivery. We included the perspectives of doctors, pharmacists, and nursers program managers, social workers, peer trainers, community staff, policymakers, volunteers, and any other stakeholders. Using the three-step meta-aggregation approach, we pool the findings of qualitative studies into three synthesized categories around the six key components: knowledge of PrEP, supportive environment, medication-related barriers, provider-level factors, financial challenges, and the model of PrEP providing service.

This review found that the knowledge about PrEP was highlighted by HCWs as an important factor related to PrEP uptake, and there was still much misinformation and misconceptions about PrEP. Previous studies have demonstrated that the knowledge was the key to addressing the gap between awareness and actual uptake of PrEP (34, 35). Only when target populations mastered the knowledge needed to better understand PrEP would they have the willingness to use it (36). According to HCWs’ reports, misinformation about PrEP was found in the following areas, misunderstanding the purpose of PrEP, excessive concerns and overstatements regarding its side effects. The misunderstanding that PrEP was used as antiretrovirals for the treatment of HIV rather than HIV prevention was the main obstacle to initiating the PrEP because of its identical appearance to commonly used oral HIV treatment tablets (37). Considering the social stigma of HIV, target populations and their parents may refuse to PrEP due to fear of being labeled HIV positive (38). We also found the excessive concerns and overstatements about the side effects to be an issue, and they mainly came from social media and were amplified by ‘fake news’ on the internet, causing their hesitation to initiate and adhere to PrEP (4). In fact, not everybody will experience side effects, and most reported side effects are transient and slight (39, 40). To address the knowledge gaps mentioned above, more promoting and health education campaigns should be conducted based on schools and communities in order to improve the conception of the potential PrEP clients and their parents or partners, and promote a positive image of PrEP users (41, 42).

This review also identified that support from parents and/or partners was another key factor influencing the willingness and persistence of PrEP uptake. To promote PrEP consumption, especially in adolescents, a supportive environment was important (17). However, due to misconceptions about PrEP, many parents may draw an unreasonable connection between PrEP uptake and HIV-positive, high-risk sexual behavior or promiscuity, causing them to disapprove of PrEP initiation for their children (43). For women, especially in the context of patriarchal society, their partners played a major role in the acceptability, use, and adherence to PrEP (44). Most women follow their partners’ instructions regardless of their own perceived or actual risk, posing a challenge to PrEP implementation (15). Therefore, we should involve their parents or partners in PrEP promotion and health education campaigns, as well as counseling before PrEP initiation, not just for the clients (17). Meanwhile, we could select some active PrEP users as ambassadors and peer supporters to disseminate information about PrEP to their peers and communities and recruit more potential target populations to participate in the PrEP program (45, 46).

Our findings indicated that some characteristics of PrEP pills may act as barriers to uptake. The effectiveness of PrEP in preventing HIV infection has been well documented, but it has also been used for HIV treatment, causing confusion between HIV prevention and treatment, which may lead clients to refuse to take it for fear of the HIV-related social stigma (47). Therefore, a different packaging or container for PrEP could be considered, and with a label indicating that is used for HIV prevention, to address the potential stigma (19). At the same time, the adherence of daily oral medication use is a challenge, and they would be more willing to have an injectable HIV prevention product rather than take daily oral tablets. Therefore, changing the daily medication into on-demand medication or long-acting injectable drugs might help to circumvent some of the issues relating to adherence and PrEP stigma (17, 48). Additionally, advocating for smaller pill sizes, as suggested by both HWCs and clients, may also improve adherence to PrEP (4, 46).

The studies included in this review also demonstrated the structural barriers at the provider level. HCWs, as the key population in the delivery of PrEP, their knowledge, attitudes, and concerns were determinants of the successful implementation of PrEP (49, 50). However, the findings showed that HCWs’ knowledge of PrEP should be strengthened, as they were unclear on how to accurately prescribe PrEP, and even failed to differentiate PrEP and PEP (30). As the gatekeepers of PrEP delivery, HCWs should be trained systematically with official policies and guidelines around PrEP/PEP uptake in medical/professional training and continuing education programs to aid in PrEP scale-up and address these knowledge gaps (30). However, there was a general perception among the HCWs in LMICs that their qualified human resource capacity was inadequate, and these providers were some of the most overburdened workers in the healthcare system (4). Therefore, it is urgent to increase the investment of human resources to promote the implementation of PrEP scale-up.

Given that we limited the included studies’ settings to LMICs, the financial challenges may be a significant barrier to PrEP scale-up. Studies have shown that many target clients live in circumstances of poverty and compete for survival, which means that other basic needs take priority over HIV prevention (4, 51). Previous studies conducted in sub-Saharan African contexts also reported that lack of funds for transport and poverty were important barriers to PrEP, and approximately two-thirds of participants were unwilling to pay for PrEP (46, 52). Therefore, from the government side, more funding should be invested to provide free PrEP, solving clients’ financial concerns. Additionally, we could eliminate the cost associated with accessing PrEP, such as reimbursement of transportation costs (32). Some HWCs suggested that economic incentives may improve PrEP initiation and adherence, however, evidence to support this practice was limited (53). From the successful experience of promoting HIV testing or ART adherence, financial incentives were effective, which can be further explored in the PrEP implementation program (54).

The findings from this review suggested that the PrEP delivery model needs to be optimized to promote PrEP implementation. In some high-income countries, such as the U.S., a variety of clinic-based, pharmacy-based, and telehealth-based models have been used for PrEP delivery (55). However, in many LMICs, PrEP delivery remains largely confined to clinics, and differentiated PrEP delivery models are needed to promote PrEP scale-up (56, 57). Integrating PrEP providing services into other healthcare systems could reduce HCWs’ already overwhelming workload. From the clients’ side, they could obtain PrEP in general clinics, pharmacies, communities and *via* mobile phone, instead of sexual health clinics, which may address their concerns about stigma and costs, and protect their privacy (58, 59). Moreover, a one-size-fits-all delivery model is unlikely to reach the large populations that could benefit from PrEP, since many potential clients at HIV risk who do not regularly frequent healthcare clinics (31).

## 5. Limitations

This review had some limitations. First, we only included studies published in English in our review, so we may have missed relevant studies from non-English speaking countries. Second, due to the purpose of our research, only qualitative studies from the perspective of HCWs were included or only data from HCWs were extracted. Evidence from clients was not included, therefore, the barriers, suggestions and demands from the clients’ side should also be considered in the implementation of PrEP. Third, although the included studies were all good quality, almost all the included studies did not report the statement locating the researcher culturally or theoretically and the influence of the researcher on the research, which may weaken the overall quality of the evidence.

## 6. Implications for future research and practice

To facilitate future PrEP implementation in LMICs and achieve the goal of ending the HIV epidemic, in future research and practice should:

- More PrEP promoting and health education campaigns should be conducted to improve the public’s awareness and address the PrEP-related social stigma.
- Focus on their parents or partners, not just the target populations, to create a supportive environment.
- Increase human resources and financial investment and eliminate structural barriers to PrEP obtaining.
- Provide a variety of delivery models to meet different clients.

## 7. Conclusion

This review integrated the experience of healthcare workers implementing prep in LMICs following the JBI’s meta-aggregation approach. We developed three synthesized categories, the barriers, facilitators, and recommendations for PrEP implementation. We could focus on the following key points to promote the uptake of PrEP, improve knowledge about PrEP, create a supportive environment, address medication related barriers, increase the human resources and financial investments, and diversify the providing models.

## Data availability statement

The original contributions presented in the study are included in the article/Supplementary material, further inquiries can be directed to the corresponding author.

## Author contributions

LZ, YS, and HC contributed to the conception and design of the study, and wrote the first draft of the manuscript. LZ, HC, XZ, and YL contributed to literature search and screening, analysis, and interpretation. All authors contributed to the article and approved the submitted version.

## Funding

This study was supported by Sichuan Science and Technology Program (Award ID: 2023YFS0045).

## Conflict of interest

The authors declare that the research was conducted in the absence of any commercial or financial relationships that could be construed as a potential conflict of interest.

## Publisher’s note

All claims expressed in this article are solely those of the authors and do not necessarily represent those of their affiliated organizations, or those of the publisher, the editors and the reviewers. Any product that may be evaluated in this article, or claim that may be made by its manufacturer, is not guaranteed or endorsed by the publisher.
