# Supplementary material for: The experience of healthcare workers to HIV pre-exposure prophylaxis (PrEP) implementation in low- and middle-income countries: a systematic review and qualitative meta-synthesis
Source: Front Public Health. 2023 Aug 24;11:1224461. doi: 10.3389/fpubh.2023.1224461 (PMC10484594; doi:10.3389/fpubh.2023.1224461)
Supplement: Supplementary file 1 [file Table_1.docx]

**Supplementary Material 1**. The search strategies

| **Database** | **Search Strategies** |
| --- | --- |
| Pubmed | #1((((((((((HIV[MeSH Terms]) OR (HIV Infections[MeSH Terms])) OR (HIV[Title/Abstract])) OR (HIV/AIDS[Title/Abstract])) OR (human immunodeficiency virus[Title/Abstract])) OR (human immuno-deficiency virus[Title/Abstract])) OR (AIDS[Title/Abstract])) OR (Acquired Immunodeficiency Syndrome[MeSH Terms]))) OR (acquired immuno-deficiency syndrome[Title/Abstract])) OR (acquired immune-deficiency syndrome[Title/Abstract])  Sort by: Publication Date |
|  | #2(((((Pre-Exposure Prophylaxis[MeSH Terms]) OR (PrEP[Title/Abstract])) OR (HIV PrEP[Title/Abstract])) OR (Pre-Exposure Prophylaxis[Title/Abstract])) OR (Preexposure prophylaxis[Title/Abstract])) OR (Pre exposure prophylaxis[Title/Abstract])  Sort by: Publication Date |
|  | #3((((((Qualitative Research[MeSH Terms]) OR (Qualitative Study[Title/Abstract])) OR (Qualitative Research[Title/Abstract])) OR (mixed method*[Title/Abstract])) OR (qualitative[Title/Abstract])) OR (interview*[Title/Abstract])) OR (phenomenology[Title/Abstract])  Sort by: Publication Date |
|  | #4 #1AND#2AND#3 |
| CINAHL | S1:TI HIV OR TI Human Immunodeficiency Virus OR TI HIV infection* OR TI Acquired Immune Deficiency Syndrome Virus  Expanders - Apply equivalent subjects Search modes - Boolean/Phrase |
|  | S2: TI PrEP OR TI Pre-Exposure Prophylaxis OR TI Pre exposure prophylaxis OR TI Preexposure prophylaxis  Expanders - Apply equivalent subjects Search modes - Boolean/Phrase |
|  | S3: TI Qualitative Study OR TI Qualitative Research OR TI mixed method* OR TI qualitative method* OR TI interview* OR TI experiences OR TI phenomenology OR TI ethnography OR TI grounded theory  Expanders - Apply equivalent subjects Search modes - Boolean/Phrase |
|  | S4: (TI Qualitative Study OR TI Qualitative Research OR TI mixed method* OR TI qualitative method* OR TI interview* OR TI experiences OR TI phenomenology OR TI ethnography OR TI grounded theory) AND (S1 AND S2 AND S3)  Expanders - Apply equivalent subjects Search modes Boolean/Phrase |
| Embase | #1 'human immunodeficiency virus'/exp  #2 hiv:ab,ti OR 'human immunodeficiency virus':ab,ti OR 'hiv infection*':ab,ti OR 'aids virus':ab,ti OR 'acquired immune deficiency syndrome virus':ab,ti  #3 #1 OR #3  #4 'pre-exposure prophylaxis'/exp  #5'pre-exposure prophylaxis':ab,ti OR prep:ab,ti OR 'preexposure prophylaxis':ab,ti OR 'pre exposure prophylaxis':ab,ti  #6 #4 OR #5  #7 'qualitative research'/exp  #8 'qualitative study':ab,ti OR 'qualitative research':ab,ti OR 'mixed method*':ab,ti OR interview*:ab,ti OR phenomenology:ab,ti OR ethnography:ab,ti OR 'grounded theory':ab,ti  #9 #7 OR #8  #10 #3AND#6AND#9 |
| Web of Science | #1 (((TI=(HIV)) OR TI=(Human Immunodeficiency Virus)) OR TI=(HIV infection*)) OR TI=(Acquired Immune Deficiency Syndrome Virus)  #2 (((TI=(PrEP)) OR TI=(Pre-Exposure Prophylaxis)) OR TI=(Preexposure prophylaxis)) OR TI=(Pre exposure prophylaxis )  #3 (((((((TS=(Qualitative Study)) OR TS=(Qualitative Research)) OR TS=(mixed method*)) OR TS=(interview*)) OR TS=(experience)) OR TS=(phenomenology)) OR TS=(ethnography)) OR TS=(grounded theory)  #4 #1AND#2AND#3 |

**Supplementary Material 2** List of findings and credibility appraisal from eligible studies (N = 122 findings)

| study | number | finding | Credibility Appraisal |
| --- | --- | --- | --- |
| Duby, Z，2023 | 1 | PrEP-related stigma | Unequivocal |
|  | 2 | Negative side-effects | Unequivocal |
|  | 3 | Hard to stick to the daily tablet dosing regimen | Equivocal |
|  | 4 | Offering PrEP as an injectable | Unequivocal |
|  | 5 | PrEP ambassadors | Unequivocal |
|  | 6 | Views of religious leaders | Unequivocal |
|  | 7 | Misinformed about PrEP | Unequivocal |
|  | 8 | Parents felt poorly consulted | Unequivocal |
|  | 9 | Parents’ lack of information | Unequivocal |
|  | 10 | Raise awareness around PrEP | Equivocal |
|  | 11 | Parental support | Equivocal |
|  | 12 | Sharing information about PrEP and the program with their parents | Unequivocal |
|  | 13 | Engage parents and community members | Unequivocal |
|  | 14 | Build relationships between AGYW and their parents | Equivocal |
| Skovdal, M，2022 | 15 | Invest in human resources | Unequivocal |
|  | 16 | Develop existing capacities | Equivocal |
|  | 17 | Train more healthcare staff on PrEP | Unequivocal |
|  | 18 | Disassociate PrEP from HIV treatment | Unequivocal |
|  | 19 | Reduce and address side-effects | Unequivocal |
|  | 20 | Reduce the size of PrEP pills | Unequivocal |
|  | 21 | Provide non-judgmental services | Unequivocal |
|  | 22 | Offer youth-relevant services | Unequivocal |
|  | 23 | Maintain confidentiality | Unequivocal |
|  | 24 | Improve knowledge about PrEP through school-based campaigns | Unequivocal |
|  | 25 | Improve communication about PrEP side-effects | Unequivocal |
|  | 26 | Mobilize and involve PrEP users | Equivocal |
|  | 27 | Directly seek out AGYW at risk | Unequivocal |
|  | 28 | Reimburse bus fares | Unequivocal |
|  | 29 | Eliminate clinical costs associated with PrEP | Equivocal |
| Omollo, V,2022 | 30 | Being non-judgmental about clients’ sexual activity | Unequivocal |
|  | 31 | Being non-judgmental about missed PrEP appointments. | Unequivocal |
|  | 32 | Fulfil AGYW’s need for confidentiality | Unequivocal |
|  | 33 | Waiting to document the visit until after the client had left | Unequivocal |
|  | 34 | Actively listening | Unequivocal |
|  | 35 | Hearing and trusting information from HCPs about what to expect while taking PrEP; | Unequivocal |
|  | 36 | Make up their own minds. | Equivocal |
|  | 37 | Take charge of their health | Equivocal |
| Mwongeli, N,2022 | 38 | The knowledge that HCWs have towards PrEP | Unsupported |
|  | 39 | Attitudes | Unsupported |
|  | 40 | Increased workload | Unequivocal |
|  | 41 | PrEP adherence | Unequivocal |
|  | 42 | Women’s partners played a major role | Unequivocal |
| Kimani, M，2022 | 43 | Inadequate preparation of HCP | Unequivocal |
|  | 44 | Lack of structural resources and support | Unequivocal |
|  | 45 | Diversified delivery models | Unequivocal |
|  | 46 | Integrating PrEP counselling and delivery in the public pharmacy system | Unequivocal |
|  | 47 | Call for community collaborations | Equivocal |
| Camlin, C.S,2022 | 48 | Enthusiasm for PrEP | Equivocal |
|  | 49 | Ambivalence about providing PrEP | Equivocal |
|  | 50 | Concerns about adhere to PrEP | Unequivocal |
|  | 51 | Fears of PrEP ‘‘failures’" | Unequivocal |
|  | 52 | "‘Moral’’ dilemma | Unequivocal |
|  | 53 | Assessing and explaining HIV risk | Unequivocal |
|  | 54 | Difficulties conveying complex adherence and stopping and restarting guidelines | Equivocal |
|  | 55 | Husbands’ refusal | Unequivocal |
|  | 56 | Parents’ refusal | Unequivocal |
|  | 57 | Safety, morale, and harmony for couples 4 | Unequivocal |
|  | 58 | Counseling 3 | Equivocal |
|  | 59 | Removing transportation and time barriers | Unequivocal |
| Bogart, L.M,2022 | 60 | Mobility of target population | Unequivocal |
|  | 61 | Transport Issues | Unequivocal |
|  | 62 | Insufficient staffing | Unequivocal |
|  | 63 | Change packaging/label/insert to address stigma | Unequivocal |
|  | 64 | Integrate PrEP provision with other healthcare services | Unequivocal |
|  | 65 | Remind PrEP users through text and phone | Unequivocal |
|  | 66 | Select adherence supporters | Unequivocal |
|  | 67 | Increase access points | Unequivocal |
|  | 68 | Provide trainings for healthcare providers and local leaders | Unequivocal |
|  | 69 | Promote PrEP through current PrEP users | Unequivocal |
|  | 70 | Promote PrEP through local community leaders/providers | Unequivocal |
|  | 71 | Use preferred media sources | Unequivocal |
| Roche, S.D，2021 | 72 | Proximal advantages Convenience | Unsupported |
|  | 73 | Privacy | Unequivocal |
|  | 74 | Autonomy | Unequivocal |
|  | 75 | Increased uptake | Unequivocal |
|  | 76 | Increased adherence | Unequivocal |
|  | 77 | Reduced HIV incidence | Unequivocal |
| Restar, A.J,2021 | 78 | Inaccurate information on the use of prep | Unequivocal |
|  | 79 | Lack of current information, prescribing recommendation and guidelines | Unequivocal |
|  | 80 | The cost associated with visits | Unequivocal |
|  | 81 | A strain on the current HIV healthcare system | Unequivocal |
|  | 82 | Risk behaviors | Unsupported |
| Lanham, M，2021 | 83 | Lack of PrEP awareness | Unequivocal |
|  | 84 | Stigma | Unequivocal |
|  | 85 | Lack of PrEP disclosure to partners and parents | Unequivocal |
|  | 86 | Intensified counseling | Equivocal |
|  | 87 | Peer support groups | Equivocal |
|  | 88 | Other HIV services that could be applied to PrEP | Unequivocal |
| Jackson-Gibson, M,2021 | 89 | The organization’s willingness | Equivocal |
|  | 90 | The use of safe spaces and linkage with the existing health care system | Equivocal |
|  | 91 | The use of peer mentors | Unequivocal |
|  | 92 | The education of male sexual partner(s) and parents | Unequivocal |
|  | 93 | The engagement of community stakeholders | Unequivocal |
|  | 94 | Continuous evaluation | Unequivocal |
|  | 95 | Limited human resources | Unequivocal |
|  | 96 | Limited financial resources | Unequivocal |
| Bärnighausen, K,2020 | 97 | Increase in sexual partners | Unequivocal |
|  | 98 | Workload | Unequivocal |
|  | 99 | Policy guidelines | Unequivocal |
|  | 100 | Prevention tools | Unequivocal |
|  | 101 | Community leaders’ influence | Unequivocal |
|  | 102 | Adolescent girl focused promotion | Unsupported |
| Pilgrim, N，2018 | 103 | Stigma and discrimination | Unequivocal |
|  | 104 | Concerns about behavioral disinhibition | Unequivocal |
|  | 105 | Lack of confidentiality | Unequivocal |
|  | 106 | Communication and information | Unequivocal |
|  | 107 | Adequate and regular training | Unequivocal |
|  | 108 | Negative impact of PrEP on existing services | Unequivocal |
|  | 109 | Integration of PrEP into comprehensive services | Unequivocal |
| Mack, N，2014 | 110 | Dearth of trained health workers | Unequivocal |
|  | 111 | Lack of equipment in laboratories | Unequivocal |
|  | 112 | Providing mobile HIV testing and counselling | Unequivocal |
|  | 113 | Incentives | Unequivocal |
|  | 114 | Establishing effective follow-up systems | Unequivocal |
|  | 115 | Community and family members' non-acceptance | Unequivocal |
|  | 116 | Stigma | Unequivocal |
|  | 117 | The mobility of target population | Unequivocal |
|  | 118 | Involve them directly in planning activities | Unequivocal |
|  | 119 | Fear of HIV testing and learning one’s status | Unequivocal |
|  | 120 | Side effects of PrEP | Unequivocal |
|  | 121 | Fear the pain of being pricked and losing a lot of blood | Unequivocal |
|  | 122 | The distance to the dispensing facilities | Unequivocal |

**Supplementary Material 3** Summary of study finding, categories, and synthesized categories to generate synthesized findings on the barriers, facilitators, and recommendations of PrEP implementation (“unsupported” findings were excluded)

| **The number of findings** | **Categories** | **Synthesized findings** |
| --- | --- | --- |
| 2, 3, 41, 50, 119, 120, 121 | Medication-related barriers | The barriers of PrEP implementation |
| 1, 84, 85, 97, 103, 104, 116 | Stigma towards PrEP |  |
| 40, 43, 44, 54, 62, 79, 81, 89, 95, 96, 98, 99, 105, 108, 110, 111 | Barriers at the level of providers and facility |  |
| 6,7, 8, 9, 49, 51, 52, 55, 56, 78, 83, 115 | Misinformation about PrEP |  |
| 60, 61, 80, 105, 117, 122 | The cost of service obtaining |  |
| 11, 12, 13, 91,92, 93 | Creating a supportive environmental | The facilitators of PrEP implementation |
| 21, 22, 23, 30, 31, 32, 33, 34, 35, 73, 74, 90, 94 | The positive experience of service acquisition |  |
| 36, 37, 48, 53, 75, 76, 77, 100 | Perceived benefit of PrEP |  |
| 5,14, 66, 87, 101 | Find support | The recommendations to facilitate PrEP implementation |
| 4, 9, 20, 63 | Change of medication |  |
| 10, 24, 25, 58, 71, 86, 106, 107 | Improve the perceptions about PrEP |  |
| 15, 16, 17, 28, 29, 59, 68, 113 | Increase human and financial investment |  |
| 18, 26, 27, 45, 46, 47, 64, 65, 67, 69, 70, 88, 109, 112, 114, 118 | Optimize the current PrEP providing service model |  |

**Supplementary material 4** ENTREQ checklist (Enhancing transparency in reporting the synthesis of qualitative research)

| **No. Item** | **Guide Questions/Description** | **Reported on Page** |
| --- | --- | --- |
| 1. Aim | This article aims to aggregate the healthcare workers’ experiences of providing pre-exposure prophylaxis in low- and middle-income countries, and find the barriers, facilitators, and recommendations of pre-exposure prophylaxis implementation. | P1 |
| 2. Synthesis methodology | Identify the synthesis methodology or theoretical framework which underpins the synthesis, and describe the rationale for the choice of methodology (e.g. meta-ethnography, thematic synthesis, critical interpretive synthesis, grounded theory synthesis, realist synthesis, meta-aggregation, meta-study, framework synthesis) | P3 |
| 3. Approach to searching | The search was pre-planned. Comprehensive search strategies were undertaken to seek all available studies. | P3; Supplementary Material 1 |
| 4. Inclusion criteria | Studies were included if they met all the following criteria: 1) Participants: according to our aim of this review, we defined the HCWs broadly, and included any participant who was directly or potentially related to PrEP implementation, doctors, nurses, pharmacists, clinical coordinators, healthcare providers, social workers, counselors, community staff, PrEP program managers and so on. 2) Phenomenon of interest: the (potential) barriers and facilitators during PrEP implementation, and recommendations improving PrEP implementation based on their experience and cognition. 3)Context: We limited the study settings to low- and middle-income countries following World Bank Country and Lending Groups. 4)Study design: qualitative research with no limitation of the methodology (e.g., phenomenology, ethnography, or grounded theory method) and the mixed method studies were included if they offered clear qualitative analysis and the primary data could be extracted.  Exclusion criteria included: 1) review articles, conference abstracts, posters, books, and dissertations, 2) the participants were HCWs plus other populations, which made it difficult to separately extract the qualitative data of HCWs separately, 3) repeated publications, 4) not available of full texts, and 5) non-English articles.Included studies used widely accepted qualitative data collection methods, with well-described methodology, including for example interviews, focus groups, direct observation, and participatory action research. Included studies also needed to have provided a clear description of recognized qualitative data analysis methods (e.g., grounded theory, narrative analysis, content analysis, thematic analysis).  Excluded studies included those for which it was difficult to extract qualitative data, e.g., mixed methods studies without clearly labeled data, or studies in settings where perceptions of parents or caregivers around infant and young child feeding could not be identified, such as summaries or aggregated data. Commentaries, protocols, and systematic reviews were not included in the analysis. | P3 |
| 5. Data sources | A comprehensive search was conducted from the databases inception to 16th March 2023 in four databases: PubMed, CINAHL Plus with Full Text, Embase, and Web of Science. The search strategy used a combination of medical subject headings (MeSH), title, abstract, keywords and Boolean calculation. Results were limited to journal articles, dissertations, theses written in English, and published before March 16, 2023. Conference proceedings, editorials, commentaries, abstracts only, newsletters, addresses, and research protocols were excluded manually. Reference lists of all selected articles were independently screened to identify additional studies left out in the initial search. Searching strategies in each database can be found in the Supplementary Material 1. | P3 |
| 6. Electronic Search strategy | Supplementary Material 1 describes the literature search | Supplementary Material 1 |
| 7. Study screening methods | The process of selection was performed by two authors (Zhang and Liu) independently, following the PRISMA guidelines (see Fig.1). All search results were imported into the reference management program Endnote X9. After removing duplicates from the primary 3220 studies, the two researchers independently screened the titles and abstracts of the studies following our inclusion criteria. We read the full text of potentially relevant studies to select the eligible articles to be included in this review and meta-synthesis, and we detailed and categorized the reasons for the exclusion of the excluded studies. Any disagreement in the selection process was resolved through a discussion between two researchers or consultation with a third researcher (Chen). | P3 |
| 8. Study characteristics | Table 1 presents the characteristics of the included studies (author(s), year of publication, country, setting, aim,sample size, methodologic & sampling approach, method of data collection and analysis, mjor theme | Table 1 |
| 9. Study selection results | A flow diagram using PRISMA guidelines for reporting of systematic reviews is presented in Figure 1 in reporting of the selection process and results. | Figure 1 |
| 10. Rationale for appraisal | A minimum of two trained reviewers (Zhang and Liu) independently evaluated the methodological rigor of the included literature following the Checklist for Qualitative Research (Critical Appraisal tools for use in JBI Systematic Reviews) (Lockwood et al., 2015). This checklist includes ten items, and each item is evaluated with “yes”, “no” or “unclear”. When the evaluation results conflicted, the third researcher (Chen), with expertise in qualitative research, decided finally. A study was included if the item of it achieved a minimum of 60% “yes” to guarantee the study showed acceptable quality. Studies were considered to possess acceptable quality if 60% of items were answered “yes”, to possess good quality if 70-90% of items were answered “yes”, and to have high quality if 100% of items were answered “yes” (Talley et al., 2021). | P3 |
| 11. Appraisal items | 2020 version of Checklist for Qualitative Research (Critical Appraisal tools for use in JBI Systematic Reviews) | P3; Table 2 |
| 1. Appraisal   process | The appraisal was conducted independently by two independent reviewers. The two reviewers discussed if consensus was required. When the evaluation results conflicted, the third researcher (Chen) decided. | P3 |
| 13. Appraisal results | Appraisal results are presented in Table 2 | Table 2 |
| 14. Data extraction | The data extraction consisted of two phases:1) extracting the general details of the study, such as citation details, and 2) extracting findings, which were defined as verbatim extracts of the author’s analytic interpretation of the results or data. During the extraction of findings, the level of “credibility” should be allocated based on the reviewers’ assessment of the degree of fit, or congruency between the data and the accompanying exemplar quote. There are three levels of credibility. A finding was rated as “unequivocal” if the congruence of the finding and the illustration accompanied was beyond a reasonable doubt, as “equivocal” if a clear association between them was lacking, or as “unsupported” if the data did not support the findings. Only unequivocal and equivocal findings were included, and unsupported findings were not presented in the synthesis result. The specific process of data extraction was conducted by the same two researchers using a predesigned Excel spreadsheet, including the first author, publication year, country, type of participants, study setting, study aim, sample size, methodology, sampling approach, method of data collection and analysis, major themes, subthemes, and primary quotes. The third researcher (Chen) verified the extracted information, and any disagreement was resolved through discussion among three authors (Zhang, Liu, and Chen) | P3-4 |
| 15. Software | Endnote X9 was used to manage the citations. | P3 |
| 16. Number of reviewers | A minimum of two trained reviewers (Zhang, Liu, Song, and Chen) | PP3-4 |
| 17. Coding | JBI meta-aggregation did not use the technique of coding | NA* |
| 18. Study comparison | The process of aggregation involves the synthesis of findings by categorizing them through the similarity in meaning. Then, we subject these categories to a synthesis to generate more comprehensive findings. | P3 |
| 19. Derivation of themes | We subjected these categories to further synthesis to generate more comprehensive findings called synthesized findings. Only unequivocal and credible findings were included. Not supported findings were not presented in the synthesis or the results (Lockwood et al., 2015). | P3 |
| 20. Quotations | “No of finding file” provides findings and quotations from the primary studies to illustrate themes and constructs, and identify whether the quotations were participant quotations of the author’s interpretation. | Supplementary Material 2-3 |
| 21.Synthesis output | Synthesis output is presented in Supplementary material 2-3 | Supplementary Material 2-3 Summary of study findings, categories, and synthesized categories to generate synthesized findings on the experience of healthcare workers in PrEP implementation in low- and middle- income countries |

^*^NA means 'not applicable'
